# Correspondence

**Published:** 1903-07

**Authors:** H. A. West

**Affiliations:** Secretary; Galveston, Texas


### Correspondence.

GALVESTON, TEXAS, July 17, 1902.

*Editor Texas Medical Journal.*

I beg through your columns to advise the members of the State Medical Association of Texas that, owing to the sickness in the family of the stenographer employed at San Antonio meeting and other unfortunate circumstances, I have not been able until the present time to obtain a transcript of the minutes of the San Antonio meeting. I mention this now in order to prepare our members for the delay in the appearance of the proceedings. The minutes, however, are now complete. Another cause of delay was the difficulty in arranging for the section officers. Several declined the appointments and their places had to be filled by nominations of the President. I will say in this connection that owing to the absence of Dr. W. R. Blalock in Europe, who was appointed councilor for the Twelfth, or Brazos Valley District, the President appointed Dr. J. J. Robert, of Hillsboro, councilor for that district.

The councilors are nearly all actively at work. A number of counties have been eager and waiting for organization. Some delay was occasioned in this work by the detention of the constitution and by-laws, which were not received until a few weeks ago. Ample supplies have since been furnished each councilor, and there is no reason now why the work of organization should not proceed rapidly and effectively. There is one point which should be made clear. I mention it here as there has been some misunderstanding in regard to it. That is, that the fee of $2.00 per member to the State Association should accompany the application in the county societies. This is not only made obligatory by Section 1, Chapter 5, in the By-Laws for County Societies, which states that the admission fee must accompany the application, but by a resolution which was adopted at the San Antonio meeting. It is not essential that the dues should be sent from the treasurer of the county societies to the Treasurer of the State Association at once, but it is just as well that this should be done; at any rate it is necessary that the secretary of the county society shall notify the Treasurer of the State Association, or the chairman of the Councilors, that the fee has been collected. I shall be glad to furnish the names of such new societies as receive charters from time to time for the information of your readers.

Fraternally yours,

H. A. West,

Secretary.

